# Comments to the Editor Re: Papukashvili et al. *Nutrients* 2020, *12*, 184

**DOI:** 10.3390/nu12071965

**Published:** 2020-07-02

**Authors:** Cyril Willson

**Affiliations:** EuSci LLC, 1309 S 204th St, #293, Elkhorn, NE 68022, USA; cmwillson@gmail.com; Tel.: +1-402-709-0336

Papukashvili et al. [1] recently covered the enzyme semicarbazide-sensitive amine oxidase (SSAO) as a potential molecular target for the management of obesity and related diseases. While the paper is interesting, there are aspects which require clarification as it relates to caffeine.

First, the authors explain in the text of the paper that caffeine demonstrated an inhibitory concentration (IC) of 0.1–10 mM with an IC_50_ of 0.8 ± 0.3 mM (Table I incorrectly uses nM for the unit of measurement) for SSAO (from bovine serum) activity, which they claim roughly corresponds to 1–4 cups of regular coffee and is consistent with a daily dose of 400 mg of caffeine. However, this is not correct. The IC_50_ for caffeine of 0.8 mM (millimolar) is well beyond the peak plasma concentration (C_max_) that humans experience after 1–4 cups of coffee or 400 mg of caffeine [2]. For example, a 100 mg oral dose of caffeine administered as coffee produced a C_max_ of approximately 2.5 mg/L, while 0.8 mM is equivalent to approximately 155 mg/L, which is nearly twice the known lethal concentration of 80 mg/L for humans [2]. Fredholm [3] noted long ago that because plasma caffeine concentrations experienced after humans ingest caffeine-containing beverages are typically below 100 µmol (0.1 mM) or around 19.4 mg/L, mechanisms explaining caffeine’s therapeutic effects should be sought in this range, while those requiring concentrations in the mM range are only of potential toxicological interest. Thus, like many potential targets of caffeine, SSAO does not appear to be relevant at non-toxic or non-lethal concentrations [2], an important distinction in order to avoid consumers being misled about the potential weight loss effects of caffeine based upon in vitro data [4].

Second, the authors indicate that caffeine is an effective agent for causing weight loss. However, the work cited, such as that by Westerterp-Plantenga et al. [5], did not provide caffeine as an intervention but rather a green tea extract to individuals who were habitual consumers of either high or low amounts of caffeine. Since the source of these subjects’ caffeine was mainly coffee, it would seem premature to conclude that caffeine alone was responsible. While caffeine itself is well known to increase lipolysis and thermogenesis, only a limited amount of the liberated fatty acids are oxidized in a resting state, leading to very limited, if any, effects upon fat mass or body weight [6,7,8]. There is some evidence that coffee or coffee extracts may produce small to modest weight and/or fat loss in humans but this would be unlikely to suffice as an actual treatment for obesity and importantly, these effects are unlikely to be due to caffeine alone [9,10].
